# Commentary on “Desialylated Platelets Maintain Immune Quiescence through Regulating Kupffer Cells”

**DOI:** 10.34133/research.0279

**Published:** 2023-12-12

**Authors:** Chen Li, Craig N. Morrell

**Affiliations:** ^1^ Aab Cardiovascular Research Institute, University of Rochester School of Medicine and Dentistry, Rochester, NY, USA.; ^2^ Department of Pharmacology and Physiology, University of Rochester School of Medicine and Dentistry, Rochester, NY, USA.; ^3^ Department of Medicine, University of Rochester School of Medicine and Dentistry, Rochester, NY, USA.; ^4^ Department of Microbiology and Immunology, University of Rochester School of Medicine and Dentistry, Rochester, NY, USA.; ^5^ Department of Pathology and Laboratory Medicine, University of Rochester School of Medicine and Dentistry, Rochester, NY, USA.

Platelets have long been studied for their roles in hemostasis and vascular injury repair. However, there is a continually evolving and deepening understanding that platelets initiate and regulate responses to tissue injury and pathogens, leading to platelets now being considered immune modulatory cells [1]. Platelets actively participate in host immune responses directly through pathogen engulfing and killing, or by regulating both innate and adaptive immune responses to infection or tissue injury [2]. Activated platelets promote inflammatory responses to tissue injury or infection by direct contact dependent interactions with immune cells and through indirect interactions via released soluble factors. However, mice that are deficient in platelets have skewed T helper cell differentiation [3] and increased vascular permeability [4], implying poorly defined roles for platelets in maintaining healthy immune homeostasis.

**Fig. 1.**
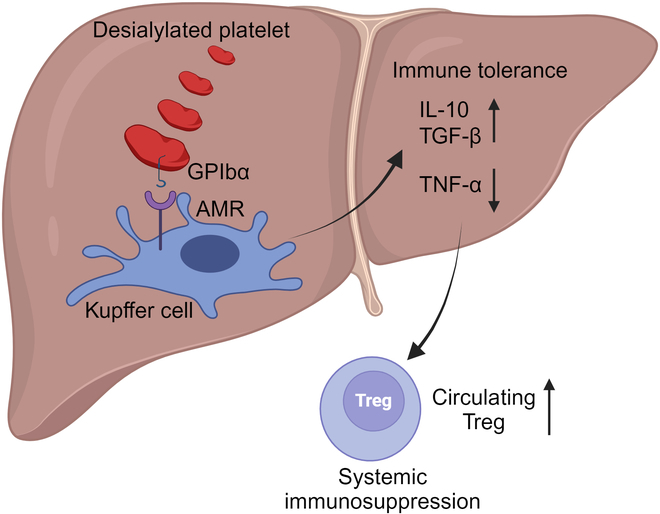
dPLT Kupffer cell.

In physiological conditions, the human body produces and removes 10^11^ platelets per day [5]. Platelets must therefore be cleared via efficient and well-regulated mechanisms. A key regulator of platelet lifespan is surface glycan modification. Platelet surface glycoprotein (GP), specifically GPIbα, is heavily glycosylated and its terminal residues are sialic acid linked to a β-galactose. Desialylated platelets (dPLTs) are recognized by the Ashwell–Morell receptor (AMR) and integrin αMβ2 on hepatocytes and/or liver macrophages (Kupffer cells, Fig. 1), leading to platelet clearance [6,7]. This is particularly important for removing senescent platelets that become desialylated as they age in the circulation. The AMR–desialylated platelet interaction on hepatocytes provides a feedback mechanism to produce TPO and maintain steady-state platelet numbers [8]. In pathological conditions, like immune thrombocytopenia (ITP), autoantibodies against platelet GPIb and CD8^+^ cytotoxic T cells can induce Fc-independent platelet activation, sialidase neuraminidase-1 translocation, and platelet desialylation, leading to platelet clearance in the liver, which can be ameliorated by the sialidase inhibitor, oseltamivir [9,10]. However, it is unclear whether there are systemic immune implications of dPLT clearance in liver.

Li et al. [11] now report that dPLT clearance in the liver stimulates an antigen-independent systemic immunosuppressive response to a secondary challenge of platelets or sheep red blood cells (sRBCs). To investigate the role of platelet desialylation in regulating immune responses, the authors transfused wild-type (WT) or dPLT into GPIbα^−/−^ or β3^−/−^ mice to induce immunoglobulin (Ig)G immune responses against platelet GPIbα or β3. Contrary to the expectation that dPLT may enhance immune responses, the authors observed significantly lower antibody titers when dPLTs were transfused to GPIbα^−/−^ or β3^−/−^ mice, compared to WT platelet transfusions. This lower antibody response was specific to platelets, as transfusion of desialylated sRBCs did not result in lower anti-sRBC IgG production. Furthermore, mice pre-sensitized/transfused with dPLTs displayed a slight, albeit significant, decrease in anti-sRBC titers after sRBC challenge. These data indicate that dPLT may act as an immune modulator and induce an immunosuppressive state that dampens more broad immune responses. This was a novel finding that implied a potentially significant role for the maintenance of platelet homeostasis in limiting immune responses.

In their studies, the authors made a novel technical advance in this research realm by utilizing photoacoustic tomography (PAT) to determine the organs that clear dPLT. PAT excites tissue in the near-infrared red region (>700 nm), allowing for deep tissue penetration, minimal photon light scattering, and signal detection by an ultrasound transducer. Combined with multispectral optoacoustic tomography (MSOT), PAT tracked indocyanine green (ICG)-coupled platelet biodistribution in real time. ICG-labeled WT and dPLTs were intravenously transfused to mice and MSOT scan revealed rapid accumulation of dPLTs in the liver and gut, while WT platelets continued to circulate throughout the whole body.

To elucidate the contribution of hepatic AMR and platelet GPIbα in targeting dPLT to the liver, the authors assessed dPLT clearance in AMR-deficient (*ASGR2*^−/−^) mice and with GPIbα^−/−^ platelets. Their data suggested that platelet GPIbα and hepatic AMR synergistically contribute to dPLT accumulation, but interactions with Kupffer cells through other mechanisms may also be involved. Kupffer cells are long-lived specialized liver macrophages that can fine-tune local and systemic immune tolerance [12]. The authors performed in vitro co-culture assays of dPLT with primary Kupffer cells to investigate how platelet clearance influenced Kupffer cell immune functions. Somewhat surprisingly, they found that dPLT dose-dependently increased Kupffer cell production of cytokines associated with reparative immune responses, including interleukin-10 (IL-10) and transforming growth factor-β (TGF-β), while decreasing tumor necrosis factor α. These findings indicated that dPLTs induced Kupffer cells to take on an immunosuppressive/reparative phenotype. It remains unclear whether desialylation itself is required for Kupffer cell immune programming, as data using in vitro co-cultures with non-desialylated platelets were not provided. TGF-β in the liver was also significantly increased in neuraminidase-treated mice, supporting the concept that dPLT liver clearance contributes to a reparative environment in the liver that may limit immune responses or drive a tissue repair and pro-fibrotic response.

T regulatory cells (Tregs) are an immunosuppressive T helper cell with its differentiation in part dependent on TGF-β. Transfusion of dPLTs, but not WT platelets, increased Tregs in the blood 3 days post-transfusion. The increase in circulating Tregs was reversed by depleting Kupffer cells, suggesting that Kupffer cell clearance of dPLT is critical for regulating Tregs. CD4^+^CD25^+^ splenic T cells (markers for Tregs) from mice transfused with dPLT displayed an enhanced suppressive function compared to non-desialylated platelet transfused mice. Furthermore, transfusion of Tregs from dPLT sensitized mice to GPIbα^−/−^ mice led to lower anti-GPIbα antibody response after platelet challenge, further suggesting suppressed T cell function in dPLT transfused mice. These data implied that dPLT interactions with Kupffer cells led to a shift in the immune environment that is more limiting to inflammation. This may be part of an immune-protective response to limit ITP or other immune-mediated mechanisms of thrombocytopenia.

While blood transfusions are common life-saving procedures, there can be adverse immune-mediated, antibody-driven, transfusion complications [13,14]. The authors transfused human dPLT into mice for 3 days prior to immunization with sRBC, in order to explore the potential for dPLT-mediated immune suppression as a means to ameliorate transfusion reactions. High-dose dPLT transfusions, but not non-desialylated platelet transfusions, significantly decreased anti-sRBC titers after sRBC challenge. Furthermore, transfusion of desialylated FVIII-expressing transgenic platelets (2bF8^Tg^) in a murine model of hemophilia (FVIII^null^) attenuated antibody responses following secondary recombinant human FVIII (rhFVIII) challenge. These studies demonstrated potential translational utility to the findings.

Taken together, this exciting work demonstrated that dPLT clearance in the liver stimulates a systemic immunosuppressive response to a secondary challenge of platelets or sRBCs. The authors found that transfused dPLT predominantly targeted to the liver, dependent on platelet GPIbα and hepatic AMR. Compared to splenic macrophages, Kupffer cells in the liver are more proficient at engulfing dPLT. Both in vitro co-culture and in vivo assays revealed that dPLTs immune program Kupffer cells to produce more immunosuppressive cytokines, including IL-10 and TGF-β, and result in an immune quiescence state with increased Tregs. These findings set the stage for future investigation into roles for platelets in maintaining local and systemic immune homeostasis. This study provides some clues that dPLT may be useful as a potential treatment to attenuate allo- or iso-immune responses following blood transfusion. It is also interesting to consider whether autologous dPLT could be used as a strategy to limit liver inflammation or transplant rejection, by inducing a more immunosuppressive liver tissue environment. These studies may represent an interesting next direction for researchers.
